# The European TauroPace™ Registry

**DOI:** 10.3390/mps6050086

**Published:** 2023-09-15

**Authors:** Reinhard Vonthein, Benito Baldauf, Stefan Borov, Ernest W. Lau, Marzia Giaccardi, Roberto Cemin, Ojan Assadian, Philippe Chévalier, Kerstin Bode, Hendrik Bonnemeier

**Affiliations:** 1 Institut für Medizinische Biometrie und Statistik, Universität zu Lübeck, Ratzeburger Allee 160, 23562 Lübeck, Germany; 2 Institute of Life Science, Hochschule Bremerhaven, An der Karlstadt 8, 27568 Bremerhaven, Germany; 3 Medical Faculty, Christian-Albrechts University, Christian-Albrechts-Platz 4, 24118 Kiel, Germany; 4 Department of Cardiology, Klinikum Freising, Alois-Steinecker-Straße 18, 85354 Freising, Germany; 5 Department of Cardiology, Royal Victoria Hospital, Grosvenor Road, Belfast BT12 6BA, UK; 6 Department of Cardiology, Ospedale Santa Maria Annunziata, Ponte a Niccheri, 50012 Florence, Italy; 7 Department of Cardiology, Ospedale Regionale San Maurizio, Bolzano, Via Lorenz Böhler 5, 39100 Bolzano, Italy; 8 Regional Hospital Wiener Neustadt, Wiener Neustadt 2700, Austria; 9 Institute for Skin Integrity and Infection Prevention, School of Human and Health Sciences, University of Huddersfield, Huddersfield HD1 3DH, UK; 10 Department of Cardiology, Hôpital Louis Pradel, 59 Bd Pinel, 69500 Bron, France; 11 Department of Electropyhsiology, Herzzentrum Leipzig, Strümpellstraße 39, 04289 Leipzig, Germany; 12 Department of Cardiology, Helios Klinikum Cuxhaven, Altenwalder Ch 10, 27474 Cuxhaven, Germany; 13 Department of Cardiology, Helios Klinikum Wesermarsch, Mildred-Scheel-Straße 1, 26954 Nordenham, Germany

**Keywords:** taurolidine, cardiac implantable electronic device, infection

## Abstract

Background: Cardiac implantable electronic device (CIED) placement comes with certain complications. CIED infection is a severe adverse event related to CIED placement. In randomised controlled trials, the preoperative intravenous administration of antibiotics and the adjunctive use of an antibiotic mesh envelope resulted in significant reduction in infections related to cardiac implantable electronic devices. The adjunctive use of taurolidine for this purpose is relatively novel and not considered in the guidelines. The required evidence may consist of a set of clinical studies. Methods: The European TauroPace^TM^ registry (ETPR) prospectively evaluates every consecutive invasive procedure involving any CIED with adjunct TauroPace™ use in the contributing centres. As the estimation of the infection rate needs to be defensible, only interventions registered prior to the procedure will be followed-up. The endpoint is a major cardiac implantable electronic device infection according to the novel CIED infection criteria (1). Secondary endpoints comprise all-cause mortality, complications, adverse events of all grades, and major CIED infections during all follow-up examinations. The follow-up times are three months, twelve months, and eventually 36 months, as acute, subacute, and long-term CIED infections are of interest. Results: As the rate of CIED infections is expected to be very low, this registry is a multicentre, international project that will run for several years. Several reports are planned. The analyses will be included in the case number calculations for future randomised controlled trials. Conclusions: The ETPR will accumulate large case numbers to estimate small event rates more precisely; we intend to follow up on participants for years to reveal possible late effects.

## 1. Introduction

CIED placement is routinely performed to treat cardiac arrhythmias and prevent sudden cardiac death. It is a frequently used therapy with approximately 1.5 million associated procedures per year [1], and usage is increasing [2,3]. Complications occur in around ten percent of patients after CIED placement [4]. Complications comprise lead dislodgement or malfunction, hematoma formation requiring revision, surgical site infections, and other issues [5,6]. The incidence of CIED infections is increasing [2]. Depending on the follow-up period and type of study, infection rates of 1.2–4.2% are reported [6,7,8,9].

Preventative measures proven to be effective include the use of an antibiotic envelope, preoperative intravenous antibiotic administration, and the use of a proper sterile technique. So far, these interventions have succeeded in reducing the above-mentioned infection rates to 0.7% within 12 months and 2.1% within three years [6,8]. Despite this improvement, there is still plenty of room to enhance performance. Antimicrobial surgical site (pocket) rinsing is currently discouraged due to a lack of evidence [1]. 

TauroPace^TM^ (TP, Tauropharm, Bavaria, Germany) is a versatile agent. It is a solution that can be used during any invasive procedure related to CIED placement (e.g., revision with the aim of upgrading, downgrading, or placing any CIED hardware, generator substitution, etc.). Taurolidine is the active agent. During its metabolism, N-methylol groups are released. They display chemicophysical activity against pathogens (i.e., direct destruction and anti-biofilm and anti-surface adherence properties) and the metabolic products/pathogenic constituents (i.e., endo- and exotoxins) released during their destruction [10,11,12].

The European TauroPace^TM^ registry (ETPR) will evaluate the ability of TP to reduce major CIED infections for 3 months, 12 months, and eventually 36 months post-procedure following CIED placement, CIED generator substitution, or upgrade, downgrade, or revision (procedure) in unselected patients (centres that use TP on all procedures) and selected patients (centres that use TP only in patients, who are at a higher anticipated risk of developing a CIED infection according to the PADIT calculator [7]).

The study will serve to record adverse events related to the procedure, the CIED, or the intervention with TP. 

Finally, this investigation will serve as a collection of complications and will be able to prospectively characterize the microbiology of localised CIED pocket infections and bloodstream infections.

## 2. Clinical Evaluation Plan

The ETPR is part of an investigator-initiated clinical development plan. The objective of this clinical development plan is to establish adjunct TP use in CIED procedures. The roadmap to that goal lists several studies to address the different research questions that will have to be answered. These follow the phases set out in the IDEAL framework [13]. 

Idea (IDEAL Stage 1): Taurolidine use is common in locking solutions and recommended in the current guidelines and consensus documents [14,15,16,17,18,19]. Taurolidine solutions are used during lung transplantations [20]. Taurolidine was researched for adjunct use during dental implantation, with a focus on how it affects the buccal microbiome [21,22]. It has shown good results when used during spinal fusion surgery [23]. Taurolidine has been used in different galenic formulations to treat infections in difficult settings [24]. Life-threatening ventricular assist device driveline infections and localised major CIED infections (pocket) were salvaged via debridement using adjunct taurolidine irrigation as a method of last resort [25,26,27,28,29]. 

**Figure 1:**
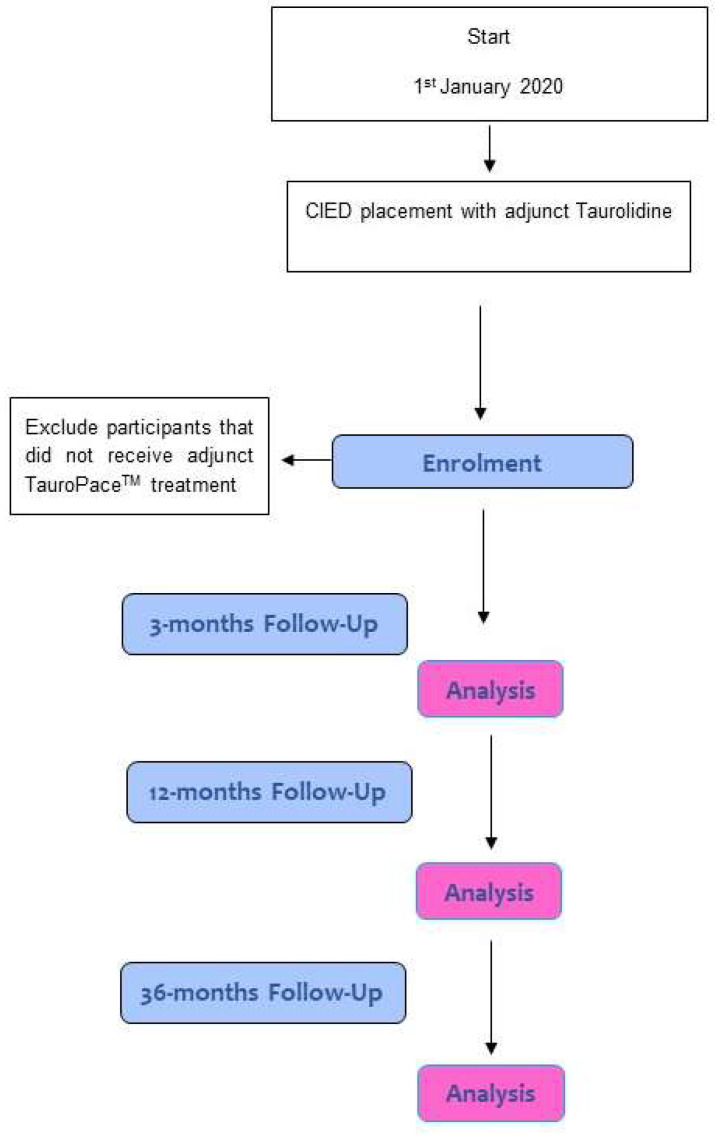
Enrolment and follow-up of ETPR participants; CIED denotes cardiac implantable electronic device.

Development (IDEAL Stage 2a): The physicians involved developed the routine use of adjunct TP during CIED placement and elaborated the standard operating procedure outlined below and on the ETPR website (www.etpr.eu; accessed on 1 June 2023). One manufacturer conducted the preclinical testing and obtained the “Conformité Européenne” for a taurolidine solution: TauroPace™. The investigator-initiated ETPR recording adjunct TP use during CIED procedures follows consecutive cases from multiple centres in a prospective manner [Figure 1]. As the estimation of the infection rate needs to be defensible, only interventions registered prior to the procedure will be followed-up. As the rate of CIED infections is expected to be very low, this registry is a multicentric, international endeavour that will run for several years (ETPR plans to include 2300 procedures and to run until 2030; this depends on the number of centres willing to participate and the annual rate of CIED procedures with adjunct TP use in these centres). Four reports are planned. Analyses will inform the case number calculations for the future studies outlined in Stage 3. The positive reporting of safety will improve the manufacturer’s post-marketing surveillance of safety issues that might otherwise be reported by employing physicians only in clinically relevant cases.

Exploration (IDEAL Stage 2b): A study researching the impact of adjunct TP use against a retrospective control gives a first impression of major CIED infection rates with and without TP (ClinicalTrials.gov Identifier: NCT05576194). These rates may be compared with comparative effectiveness measures from randomised controlled trials (RCTs) of other interventions used for the same purpose. The results could be used for a case number calculation for the planning of future studies. A case–control study of CIED extraction will investigate the reasons for CIED removal. Controls with similar risk factors for CIED infection undergoing CIED placement will be matched to the cases eligible for CIED hardware removal in the event of CIED infection. This is the only study with enough CIED infections to allow multivariable modelling of causation. Its results could guide the participant selection of an initial RCT in selected patients and the case number calculation of an ultimate RCT in unselected patients. 

Assessment (IDEAL Stage 3). Treatment guidelines may mention potential infection prevention with taurolidine after a first RCT of TP against the standard of care as defined in the treatment guidelines at that point in time. This may be the FederaL University of Schleswig-Holstein’s CIED Irrigation Trial (FLUSH-IT) with its comparator of no disinfection of the CIED and its components, or the Worldwide Antibiotic Stewardship in CIED Infection Prevention Trial (**WASH-IT**), which compares the intervention with adjunct TP, without preoperative intravenous antibiotic administration versus the standard of care at that point. To keep the size (and cost) of such a project reasonable, participants are selected according to their potential to benefit from the treatment, and the primary endpoint is defined to prove the direct effect. 

Long-term (IDEAL Stage 4). The ETPR begun at stage 2a will accumulate large case numbers to estimate small event rates more precisely; the plan aims to follow up on participants for years afterwards to reveal possible late effects. At the same time, results from the controlled cohort study and a matched case–control study, plus multiple interim analyses of the registry, are needed for an eventual RCT to begin. The prospective registry should therefore continue for the surveillance of established techniques (SOP from www.etpr.eu). 

## 3. Scope

In addition to the scope of generating feasibility data according to the clinical evaluation plan outlined above:

The ETPR should define and develop patient-, CIED- and procedure-specific understandings of factors driving complications related to the placement of CIEDs in Europe.

The ETPR may shape the development of guidance on how to reach and retain the best possible practice in CIED placement in different settings and countries across Europe.

The ETPR will develop and evaluate a set of suitable tools, solutions, and strategies applicable to the placement of different types of CIEDs with the adjunct use of a new medical device containing taurolidine.

The ETPR will build new site capabilities and develop training activities to increase the number of contributing sites and expand the pool of investigators, including investigators from under-represented communities and naïve investigators in geographies where the study infrastructure may be insufficient.

Ultimately, the ETPR will gain planning data related to the feasibility of conducting future RCTs researching the safety, clinical effectiveness, and cost-effectiveness of adjunct TauroPace^TM^ use against current standard of care in patients eligible to undergo any CIED procedure with the placement of any CIED.

## 4. Methods

### 4.1. Design

The study plan was designed by the authors (S.B. and B.B.) to meet the criteria of a prospective observational study. The observation was initiated in one contributing centre after consent from the review board of the Bavarian state medical chamber (Nr.:19059). With an increasing number of centres using adjunct TP throughout Europe for CIED placement, the initial study plan was redesigned (R.V., B.B., H.B., S.B., and O.A.) to a multi-centre observational registry (IRB CAU D420/21) and passed the competent independent ethics committees at the respective contributing centres (EC BLAEK mb21038; EC AEKN GRAE133/2022).

### 4.2. Inclusion/Exclusion

The present prospective observational study includes all consecutive patients with adjunct TP use during CIED placement. This may be in unselected patients (centres that use TP in all patients) or in patients with an anticipated risk for CIED infection according to the results of the PADIT calculator [7] (centres that use TP in high-risk patients only). CIED placement procedures include any invasive CIED procedure (i.e., revision, upgrade/downgrade with or without lead placement, or generator substitution) with adjunct TP treatment of all the CIED hardware before, during, and after placement in patients eligible to undergo a CIED placement procedure receiving any CIED (including and not limited to PPM, ICD, CRT-P, CRT-D, S-ICD, and CCM).

Every procedure with TP enters the final analysis. Standard of care comprises inspection, electrocardiogram, laboratory testing, and cardiac ultrasound for placement and a CIED interrogation in the event of generator replacement, upgrade or downgrade revision, and early revision. Additional medical examinations and diagnostics are performed according to medical needs (i.e., computed tomography angiography, vascular ultrasound, Holter electrocardiogram, coronary computed tomography, cardiac magnetic resonance imaging).

An inability to sign the patient informed consent will lead to the patient’s exclusion.

### 4.3. Intervention: Surgical Procedure and Infection Prevention Measures

All procedures are performed in an electrophysiology ward or an operational theatre with laminar airflow in the ceiling and restricted access during the procedure (no OR traffic).

➢ **Pre-procedural (intervention):**

The participant’s torso, legs, and arms should be fixed to the table. If necessary, chest hair should be clipped. The use of an external defibrillator and the recording of an intraprocedural electrocardiogram represent the standard of care. Intravenous antibiotic prophylaxis with a cephalosporin within 60 min or vancomycin within 120 min is mandatory. Antiseptic skin preparation is performed with a polyvinylpyrrolidone-iodine (PVP-I) or chlorhexidine (CHX) wash according to the centre’s standard of care, including the chest wall, both shoulders, the upper arms, and the neck. The use of polypropylene adhesive drapes and gowns and double gloving are encouraged in the contributing centres. Patients are either operated on under conscious sedation with intravenous/oral benzodiazepine or no sedation, except in cases of the implantation of a subcutaneous defibrillator (S-ICD), where general anaesthesia may be performed.

➢ **Procedure (intervention) with adjunct TP:**

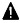
For de novo, upgrade/downgrade revision, early revision, or generator exchange and further clarification, please refer to the SOP CIED placement or SOP CIED revision in the Supplementary Materials section of this manuscript and on www.etpr.eu.

*De novo placement:* After the subcutaneous administration of local anaesthesia, the skin is incised. The generator pocket is prepared. After the placement of the sheath, the pocket is rinsed with TP. The first lead is wiped down and the suture sleeve mobilised with a TP-soaked swab. After placement, the fixation sleeve surrounding the lead is moved to its final position by gripping it with a TP-soaked swab. When sutured to the tissue, the lead, sleeve, and suture are irrigated with 5–10 cc of TP. Subsequent lead placements are carried out accordingly. Before connecting the leads to the generator box, the generator is wiped down with or fully immerged in TP. Both the lead and the generator are gripped with a TP-soaked swab when plugging the lead into the port. The torque wrench is immerged in TP before engaging with the set screws. Once all the hardware is in its final position, the pocket is rinsed with the remainders of the TP. The wound is closed. 


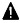
For de novo, upgrade/downgrade revision, early revision, or generator exchange of S-ICDs and further clarification, please refer to the SOP S-ICD in the Supplementary Materials section of this manuscript and on www.etpr.eu.

*Generator exchange, CIED upgrade or downgrade, any revision:* The skin of the pocket is incised cranially (avoiding skin incision “covering” the CIED). After gaining access to the generator pocket and before mobilizing the indwelling generator, the pocket is irrigated with TP (a substantial percentage of the indwelling generators may be contaminated with dormant bacteria [30]), which helps with mobilisation. The old generator is extracted from the pocket. The generator is disconnected. In the cases of substitution and upgrade revision (and a new lead in the case of an upgrade revision), the new generator is washed and wrapped in a TP-soaked swab. Then, the leads (after placement in the event of an upgrade revision) are subsequently connected to the new generator using a TP-soaked swab to grip the leads to plug them into the ports. Before engaging with the seals covering the set screws, the new torque wrench is immerged in TP to prevent contamination. Before positioning the new CIED generator, the empty fibro-collagenous pocket with the old leads in place is repeatedly rinsed with TP (5 cc each). After the final placement of the generator, all hardware is irrigated with TP prior to wound closure. “Breaking” the pocket (the induction of bleeding inside the pocket is hypothesized to trigger a humoral immune response to the possible contamination of the pocket; however, this brings with it the risk of inducing a hematoma) is at the operator’s discretion but is not recommended [1].

Other measures to prevent CIED infection, like additional pocket irrigation with antibiotics or antiseptics or the use of the antibiotic mesh envelope, are discouraged during the procedure in the contributing centres (when TP is used, no oxygenising agent like iodine or chlorhexidine should be used in the pocket or on the CIED).

➢ **Postprocedural:**

The surgical site is cleaned with PVP-I- or CHX-soaked swabs after skin closure. A sterile surgical dressing with an additional pressure dressing (including a sandbag or special bandage where available) is applied. Before the patient is discharged from the hospital, the wound is inspected and the CIED parameters are tested. Participants are trained to observe the surgical site and instructed not to take a full body bath for the following 10 days and not to swim, carry heavy loads, or exercise for the following six weeks. When showering, participants are trained not to moisten the surgical site and to use a waterproof wound dressing, which is given to them upon discharge from the hospital.

### 4.4. Termination

Participants can terminate their participation in the study at any time without providing reasons. The participants may freely withdraw consent at any time without creating any disadvantages for patient-centered care.

Some potential reasons for the premature termination of the study are the identification of unacceptable risks, or incidents leading to an unacceptable benefit–risk assessment.

### 4.5. Data

The registry allows for a full survey. The data model is based on the following data sources: primary center-based data collection e.g., on specific indication criteria and on the indication of the product under investigation (comorbidities). Indication criteria comprise patient-related risk factors (<65 years, male gender, renal impairment, COPD, heart failure, chronic skin disease, malignancy, immunosuppression, oral anticoagulation, dual platelet inhibition, diabetes mellitus, hematoma formation, and previous implant infection) and procedure-related risk factors (procedure duration longer than 59 min, inexperienced operator, revision, generator substitution, abandoned lead, more than two leads placed, temporary pacing, and bulkier/heavier devices: S-ICD, ICD, CRT), which are recorded. 

## 5. Endpoints

The primary endpoint is major CIED infection during follow-up.

Major CIED infections are defined in the novel CIED infection criteria [1], resulting in [8]:

- CIED system removal;
- an invasive CIED procedure (pocket revision without removal);
- surgical site infection resulting in pocket revision or CIED extraction after systemic antibiotic therapy;
- chronic antibiotic therapy due to a major CIED infection without an invasive procedure;
- death.

Secondary endpoints include all-cause mortality, major CIED pocket infection, minor CIED infection, TP-related adverse device effects (ADE), CIED-procedure-related or CIED-hardware-related adverse events (e.g., pain, arrhythmia, hematoma formation that is resolved, pneumothorax that is resolved), and complications (e.g., hematoma formation requiring revision, pericardial effusion requiring drainage, pneumothorax requiring chest tube, events requiring early pocket revision) during all follow-up periods.

### 5.1. Endpoint Assesment

General: Participants will be trained to be aware of the clinical criteria as defined in Section 4. Participants will be told to call whenever the slightest signs of the defined criteria are noted or in any case of unusual or suspect indisposition. If the call indicates CIED infection, adverse events, or incidents, the participant calling will be examined in the ward by a cardiologist or cardiac surgeon with expertise in rhythm surgery. A decision on the presence of a CIED infection, adverse event, or other incident will be made according to the examination results and the clinical criteria defined.

If an infection is suspected, the initial tests include (but are not limited to) an examination (inspection, temperature, questioning), trans-thoracic echocardiography (TTE), trans-oesophageal echocardiography (TEE/TOE), a complete blood count, and repeat blood cultures (at least three sets taken from different sites; blood sampling from any central venous line is prohibited). In case of hardware exposure, re-intervention, or extraction, swabs and tissue samples of the pocket interior and hardware are sent to the local microbiology lab for analysis.

### 5.2. Follow-Up Schedule

Table 1 contains the follow-up schedule after the procedure.

## 6. Statistical Analysis

The ETPR is designed for comparisons between the intervention with TP and historical controls, with a two-sided confidence level of 0.95. The primary estimand is the rate of patients who develop a CIED infection after a CIED procedure with adjunct TP before a repeat procedure (i.e., system modification) on that CIED is performed or the patient succumbs. This rate of infection is needed to evaluate the intervention with TP during CIED placement procedures. Exact binomial statistics will be used for CIED infection rates at 3 and at 12 months, estimated as the number of procedures followed by a CIED infection relative to the number of registered procedures with at least the respective follow-up times or an event within the specified time periods. For the first sensitivity estimand, the intercurrent events (Table 2) of generator replacement, system change, revision (i.e., any procedure that touches the CIED within the follow-up period), and death are considered to be competing risks in the estimation of the cumulative incidence curve. Event times end at these events, as these events can trigger CIED infections, but sub-distribution hazards are used to calculate the cumulative incidence function. This leads to fewer upward-biased estimates than when considering these intercurrent events as censoring the event time. Censoring is restricted to failure to follow-up (i.e., death, discontinuation) and the end of observation (i.e., system modification). Other intercurrent events, such as the intravenous administration of antibiotics (e.g., in the treatment of pneumonia), will not be considered, as in the published intention-to-treat data. Their sum is expected to be independent of TP use. This decision is taken to avoid estimates that are too low for the evaluation of the intervention.

Another objective is to gather planning data for future studies. In randomised controlled trials (RCT), the primary estimand is the cause-specific hazard ratio. This translates into censoring at the occurrence of competing risks. The second sensitivity estimand of the ETPR is the rate of major CIED infections per time survived without competing risks. 

The confidence level will not be adjusted for multiple checks, as all analyses will be performed, and conclusions will be based on the latest results of the interim analyses or final results only. Times to major CIED infection or to death will be analysed using Kaplan–Meyer curves and cause-specific hazard ratios for the numbers of risk factors related to the patient, the CIED, or the procedure. ADE and complications will be analysed similarly using logistic regression.

The secondary estimand is all-cause mortality after CIED procedures using TP between procedures accessing the pocket. Intercurrent events are handled similarly (see Table S1 in the online supplement).

To facilitate causal inference in future research, the known risk factors for major CIED infection are reported. Their distribution should enable the calculation of propensity scores or more general balancing weights [31]. Such analyses would consider the randomness of historic controls instead of using historic results as performance goals.

### Sample Size

The performance goals for the rate of major CIED infections vary according to country, evidence level, CIED type, and patient selection. They are expected to be higher in a revision procedure involving a complex CIED in high-risk patients than for de novo implants in otherwise healthy patients within a well-maintained setting. Additionally, the novel criteria for major CIED infection have evolved [1]. Accordingly, the published 12-month rates range from 0.7% to 4.2% (Table 3). The three-month rates are available from WRAP-IT and the Danish device registry, with values of 0.8% and 1.0% in controls, respectively [32]. For future studies researching TP use, we expect to plan for a 12-month rate of 0.5%. If its ratio to the three-month rate is 0.8/1.2, as in the WRAP-IT controls [8], then we assume a three-month rate of 0.33%.

For an approximate 95% confidence interval, to exclude a risk difference of 0.8% − 0.33% = 0.47%, when the true event rate is 0.33%, the sample size needs to exceed 572. 

For an exact 5% binomial test to have a power of 80% in detecting the difference between the hypothetical rate of 1% (0.8%) and the true rate of 0.33%, the sample size needs to be 1140 (2000).

Assuming a major CIED infection rate in the registry of 0.5% at 12 months and 0.33% at 3 months, a comparison with the performance criteria from the literature would require the sample sizes shown in Table 3.

We therefore plan to publish the ETPR results after the twelve-month follow up of 400, 900, 1420, and 2300 procedures. For the long-term evaluation, a report after the follow-up period of 36 months is planned for all procedures enrolled.

## 7. Discussion

Infection is a major risk and a clinically significant complication after CIED placement. Despite great efforts to reduce the risk of infection, the current epidemiological data indicate that prevalence rates vary but remain a constant problem [9,35]. Certain patient groups have a much higher risk of developing CIED infection due to procedure-, device-, or host-specific risk factors [34]. Recent publications have led to a better understanding of the true incidence of CIED infections, their prevalence, and underlying pathogens [6,8,36]. A proof of concept to address the surgical site, with the aim of preventing CIED-related infections, has led and is still leading to the broad use of various agents with [6,8,37], without, or despite existing evidence [7,38]. To further address and to better understand reactions and infections related to indwelling foreign bodies, investigations like the ETPR are needed. The undertaking of a clinical investigation of a marketed device makes the undertaking of a study easier and yields results that are, in our opinion, more applicable. The ETPR consecutively enrols all patients undergoing CIED placement, upgrades, downgrades, revision, or substitution. It was designed to evaluate and follow up on these procedures using a novel antimicrobial CIED irrigation solution: TauroPace^TM^. The value of this observation comes from short-term, medium-term, and long-term follow-up observations, as CIED infections are time-dependent problems and are not limited to the days following the CIED procedure. The prevention of CIED infection over the course of time, possibly related to the use of TP, is illustrated when one compares CIED infection rates from ETPR with the respective figures from the literature. 

So far, the evidence related to CIED infection prevention is limited to the use of pre-operative intravenous antibiotics and the adjunct placement of an antibiotic mesh envelope during CIED placement in a subset of patients with a high anticipated risk of CIED infection [6,8]. The use of antiseptic or antibiotic solutions in different galenic formulations used to irrigate the surgical site during CIED placement has not resulted in a significant reduction in major CIED infections in randomised controlled trials and is therefore not recommended in the current guidelines [39]. However, premature data for adjunct TP use have shown it to be promising in a large observational study [40]. Additionally, it has been used for the treatment of infection in different cardio-thoracic settings, without the risk of inducing antimicrobial resistance [25,26,27,28,29].

A beneficial effect of TP use during CIED placement is clearly driven by a low incidence of major CIED pocket infections, which account for up to two thirds of all major CIED infections and are mainly attributed to the contamination of the surgical site (CIED pockets, fibrous capsules in the event of generator substitutions, CIED hardware) at the time of CIED placement [30]. The risk of developing a pocket infection early after a CIED procedure is significant and can vary depending on the procedure, the CIED, and the follow-up time. However, CIED placement itself comes with a certain lifetime risk for infection [6]. Therefore, only a follow-up span of years can reflect the plausibility of the specific treatment benefits of TP. 

Since the adjunct use of TP, which comes as a liquid solution, involves crucial steps during the CIED procedure, the procedure time and hardware- and procedure-related adverse events will be recorded and compared with the citable literature.

Every patient eligible for CIED placement displays comorbidities and is at a higher risk of succumbing to the underlying disease when compared to the general population; therefore, documentation of all-cause mortality during all follow-up periods is good clinical practice, according to our understanding.

In order to ensure the applicability of the microbial solution containing taurolidine and the recommendations for the intervention of placing a CIED, there is a particular need for follow-up assessments of widespread use, based on the availability of contributing centres and procedures within a project like the ETPR. The proposed disease area represents an unmet public health need—especially due to increasing procedure rates in the European Union area—and, in the event of complications, a significant burden for patients, healthcare systems, and society. The proposed area, the patients to be evaluated, and the associated complications are representative, to allow for a wide implementation in different cultural and geographical distributions in the context of the proposed clinical research on our catalogue of measures, as well as on TP. Regulators, health technology assessment bodies, and payers benefit from better information on health technologies’ benefit–risk profiles across the patient populations for use in clinical practices.

In comparison to studies of similar or different characters, the ETPR is designed to evaluate multiple baseline characteristics related to the patient, the CIED, and the procedure in different healthcare systems across the continent. At the same time, it does not constrain the investigator to recruit according to certain eligibility criteria. Instead, as stated above, the recruitment of all patients/procedures is encouraged. Therefore, in addition to researching the expressed endpoints, the ETPR opens the possibility of acquiring fine-grain data regarding CIED placement rates, comorbidities, and preferred practices in a scientifically underrepresented population.

Some limitations should be considered when interpreting the potential results of the ETPR in the future: the ETPR is a prospective observational open-label study. The participants may be selected according to an anticipated risk for CIED infection as some centres might use the TP adjunct only in high-risk procedures with high-power devices and numerous host-related risk factors. As the risk calculation is undertaken according to the PADIT calculator [7], which has reliably calculated the anticipated risk for CIED infection [41], this should lead to a higher percentage of patients/procedures with an anticipated high risk for CIED infection being enrolled and should therefore support our hypothesis.

The contributing centres’ personnel might display differences in terms of expertise and the standard of care (i.e., TP use as stated above, operational theatre vs. catheter laboratory, cloth gowns and drapes, etc.).

In summary:

- The ETPR is the first registry across the European continent to research CIED placement practices, patients eligible for CIED placements, and complications in relation to the described procedures.
- The aim of the ETPR is to acquire planning data for the feasibility of conducting a future RCT, researching the safety, clinical effectiveness, and cost-effectiveness of adjunct TauroPace^TM^ use against the current standard of care in patients eligible to undergo any CIED procedure with the placement of any CIED.
- It is designed as a prospective, multi-centre, international clinical cohort study.
- It is planned to run until 2030 and to recruit at least 2300 consecutive procedures with adjunct TauroPace^TM^ use.
- The endpoints are CIED infection (minor, major, pocket-related, and lead-related infections) within 3 months, 12 months, and, eventually, 36 months, adverse events of all grades, and all-cause mortality

## 8. Conclusions

Recently, we have learned about the practice whereby the majority of centres use irrigation solutions containing various antimicrobial substances in everyday practice in the hope of gaining the advantage in the battle against localised CIED infections [38]. However, high-quality evidence to support these measures is missing or not supportive [7,39].

The solution researched in the ETPR is the use of an adjunct during CIED placement. It has been designed to keep all CIED system components disinfected during any invasive CIED procedure. It is certified in this context. The ETPR will be fit to provide information regarding efficacy while researching the safety of the intervention with TP during CIED placement, both for the patients and the CIEDs. Eventually, the ETPR will serve as a collection of complications related to the placement of CIEDs during a maximum follow-up period of 36 months.

## Figures and Tables

**Table 1:** Follow-Up Schedule.

Post-Procedure Visits	De Novo (Including Lead Only in Upgrade or Downgrade)	Box Exchange
Hospital discharge	**X**	**X**
1 month	X	
3 months	**X**	**X**
12 months	**X**	**X**
24 months	**X**	**X**
36 months	**X**	**X**

X, clinical routine; **X**, registry follow-up recorded.

**Table 2:** Intercurrent events, their handling in the analysis for the first sensitivity estimand, and the rationale. The prevented bias is the bias that would have been incurred without action. The possible bias is a bias that may be incurred by the action. CI = confidence interval, MNAR = missing not at random, MAR = missing at random.

Intercurrent Event	Prevented Bias	Action	Analysis Strategy	Missingness Assumed	Possible Bias
Other or no disinfectant	Upward	No follow-up	Principle stratum	MNAR	Downward
No permanent implantation	Downward	No follow-up	Principle stratum	MNAR	Upward
Conversion to open surgery	Upward	Full follow-up	Treatment policy	--	Upward
Antibiosis for infections not related to CIED	?	Full follow-up	Treatment policy	--	?
Pocket hematoma, septic thrombophlebitis	?	Full follow-up	Treatment policy	--	?
Minor CIED infection	?	Full follow-up	Treatment policy	--	?
Box exchange	Upward	Competing risk	Hybrid	MNAR	Wide CI
System modification	Upward	Competing risk	Hybrid	MNAR	Wide CI
Other intervention touching the CIED pocket	Upward	Competing risk	Hybrid	MNAR	Wide CI
Death	Upward	Competing risk	Hybrid	MNAR	Wide CI
Lost to follow-up for another reason	Upward, narrow CI	Censoring	Treatment policy	MAR	Wide CI

**Table 3:** Historic major CIED infection rates and sample sizes N needed to demonstrate superiority over such external fixed performance goals, if the true new rate is 0.5% at 12 months or 0.33% at 3 months. Sample sizes in bold are selected to issue registry reports.

Source	Follow Up (Months)	Historic CIED Infection Rate (Literature)	Hypothetical CIED Infection Rate (ETPR)	N for Approximate 95% Confidence Interval	N for Exact Test with Power 80%
WRAP-IT [8] verum	3	0.45%	0.33%		
WRAP-IT [8] verum	12	0.7%	0.50%	4778	12200
WRAP-IT [8] control	3	0.80%	0.33%	**572**	2000
WRAP-IT [8] control	12	1.2%	0.50%	**391**	**1420**
PADIT [7]	12	1.03%	0.50%	**681**	**2300**
Henrikson [33]	3	1.0%	0.33%	282	1140
Danish registry [32]	6?	1.43%	0.50%	221	**880**
FLUSH-IT control	12	2.4%	0.50%	53	330
US insurance [9]	NR	4.2%	0.50%	14	101
Polyzos low [34]	NR	1.0%	0.33%	282	1140
Polyzos low [34]	NR	1.0%	0.50%	765	2370
Polyzos high [34]	NR	1.3%	0.33%	135	608
Polyzos high [34]	NR	1.3%	0.50%	299	1050

NR = not reported.

## Data Availability

The study protocol, database information, and patient informed consent are available upon request at www.etpr.eu.

## Supplementary Materials

The following supporting information can be downloaded at: https://www.mdpi.com/article/10.3390/mps6050086/s1, Table S1; versions of standard operating procedures in English, Italian, and German; study protocol, patient informed consent and a database description can be downloaded at www.etpr.eu.
