# Supplementary material for: The European TauroPace™ Registry
Source: Methods Protoc. 2023 Sep 15;6(5):86. doi: 10.3390/mps6050086 (PMC10514882; doi:10.3390/mps6050086)
Supplement: Supplementary file 1 [file mps-06-00086-s001.zip › mps-2536563-supplementary.pdf]

Supplementary Materials

Table S1: Intercurrent events, their handling, and the rationale in the analysis of all-cause mortality after CIED procedures using TP between procedures touching the pocket. The prevented bias is the bias that would have been incurred without action. The possible bias is a bias that may be incurred by the action. CI = confidence interval, MNAR = missing not at random, MAR = missing at random.

Intercurrent event	Prevented bias	Action	Analysis strategy	Missingness assumed	Possible bias
Other or no disinfectant	Upward	No follow up	Principle stratum	MNAR	Downward
No permanent implantation	Downward	No follow up	Principle stratum	MNAR	Upward
Conversion to open surgery	Upward	Full follow up	Treatment policy	--	Upward
Antibiosis for infections not related to CIED	?	Full follow up	Treatment policy	--	?
Pocket hematoma, septic thrombophlebitis	?	Full follow up	Treatment policy	--	?
Minor CIED infection	?	Full follow up	Treatment policy	--	?
Box exchange	?	Competing risk	Hybrid	MNAR	Wide CI
System modification	Upward	Competing risk	Hybrid	MNAR	Wide CI
Other intervention touching the CIED pocket	Upward	Competing risk	Hybrid	MNAR	Wide CI
Major CIED infection	Upward	Competing risk	Hybrid	MNAR	Wide CI
Lost to follow up	Upward, narrow CI	Censoring	Treatment policy	MAR	Wide CI
